# Nasal high flow therapy in very low birth weight infants with mild respiratory distress syndrome: a single center experience

**DOI:** 10.1186/s13052-017-0438-9

**Published:** 2017-12-28

**Authors:** Antonio Di Mauro, Manuela Capozza, Sergio Cotugno, Silvio Tafuri, Francesco Paolo Bianchi, Federico Schettini, Raffaella Panza, Nicola Laforgia

**Affiliations:** 1 Neonatology and Neonatal Intensive Care Unit, Department of Biomedical Science and Human Oncology, “Aldo Moro” University of Bari, Policlinico Hospital - Piazza Giulio Cesare n. 11, 70124 Bari, Italy; 2 Section of Hygiene, Department of Biomedical Science and Human Oncology, “Aldo Moro” University of Bari, Bari, Italy

**Keywords:** Nasal high flow therapy, Very low birth weight infants, Neonatal respiratory distress syndrome

## Abstract

**Background:**

Pulmonary disorders and respiratory failure represent one of the most common morbidities of preterm newborns admitted to neonatal intensive care units (NICUs). The use of nasal high-flow therapy (nHFT) has been more recently introduced into the NICUs as a non-invasive respiratory (NIV) support.

**Methods:**

We performed a retrospective study to evaluate safety and effectiveness of nHFT as primary support for infants born < 29 weeks of gestation and/or VLBW presenting with mild Respiratory Distress Syndrome (RDS).

The main outcome was the percentage of patients that did not need mechanical ventilation. Secondary outcomes were rate of bronchopulmonary dysplasia (BDP), air leaks, nasal injury, late onset sepsis (LOS), intraventricular hemorrhage (IVH), retinopathy (ROP), necrotizing enterocolitis (NEC), hemodynamically-significant patent ductus arteriosus (PDA) and death.

**Results:**

Sixty-four preterm newborns were enrolled. Overall, 93% of enrolled patients did not need mechanical ventilation. In a subgroup analysis, 88.5% of infants < 29 weeks and 86.7% of infants ELBW (< 1000 g BW) did not need mechanical ventilation.

BPD was diagnosed in 26.6% of preterms enrolled (Mild 20%, Moderate 4.5%, Severe 1.5%). In subgroup analysis, BPD was diagnosed in 53.9% of newborns with GA < 29 weeks, in 53.3% of ELBW newborns and in 11.1% of small for gestational age (SGA) newborns.

Neither air leaks nor nasal injury were recorded as well as no exitus occurred. LOS, IVH, ROP, NEC and PDA occurred respectively in 16.1%, 0%, 7.8%, and 1.6% of newborns.

**Conclusions:**

According to our results, n-HFT seems to be effective as first respiratory support in preterm newborns with mild RDS. Further studies in a larger number of preterm newborns are required to confirm nHFT effectiveness in the acute phase of RDS.

## Background

Pulmonary disorders and respiratory failure represent one of the most common morbidities of preterm newborns admitted to neonatal intensive care units (NICU) [1].

Over the previous decades, mechanical ventilation (MV) has been a common practice in post-delivery respiratory care and has significantly improved the survival of preterm newborns [2]. However, the association between MV and lung injury has been well documented [3].

Therefore, the interest in non-invasive respiratory support has surged, with the aim of reducing the risk of lung injury and the incidence of BPD [4, 5].

Continuous positive airway pressure (CPAP) is the current standard of care for non-invasive respiratory support in very preterm newborns over the immediate postnatal period, when endotracheal intubation is not needed [6].

The use of nasal high-flow therapy (nHFT) has been more recently introduced into the NICU setting with several mechanisms of action proposed [7].

Due to a simple interface and small tapered prongs, nHFT is perceived as easier to use by nursing staff [8], more comfortable for the newborn [9] and advantageous for mother–newborn bonding [10–12], when compared to CPAP.

A recent meta-analysis on effectiveness and safety of nHFT in preterm newborns concluded that it is not different compared to other conventional modes of non-invasive respiratory support [13], but point out that the evidence for efficacy of nHFT in the extreme preterm infants, compared with other modes of NIV, is still poor and needs to be addressed in larger studies.

nHFT can be used in different clinical situations, including primary support in post-delivery respiratory care and as a mode of weaning from either mechanical ventilation or CPAP [14] .

A recent Cochrane Review stated that the vast majority of evidence available concerns the use of nHFT as post-extubation support and that very few trials have included extremely preterm newborns so far [15].

The aim of our study was to evaluate the effectiveness and safety of high-flow nasal cannulae therapy as primary respiratory support for infants born < 29 weeks of gestation and/or VLBW presenting with mild Respiratory Distress Syndrome (RDS).

## Methods

Since 2014, in the NICU of Department of Biomedical Science and Human Oncology, University of Bari “Aldo Moro”, Italy, adopted a protocol in which nHFT (Vapotherm® Precision Flow) substituted nCPAP as the primary mode of non-invasive respiratory support for preterm newborns. NHFT failure was considered as the need of intubation and mechanical ventilation.

This retrospective study shows the results from January 2014 to December 2016 and data were gathered from medical records.

The aim of the study was to evaluate the success rate and the safety of nHFT in our cohort of VLBW and ELBW preterm newborns.

The local Ethics Committee (Comitato Etico Azienda Ospedaliera Policlinico di Bari) approved the study protocol.

Newborns were eligible for the study if they met the following inclusion criteria:

1. Inborn
2. Gestational age < 29 weeks and/or birth weight ≤ 1500 g;
3. nHFT as first respiratory support for mild Respiratory Distress Syndrome (RDS), defined by the presence of one or more of the following within 1 h of birth:

- Silverman score ≥ 5;
- FiO_2_ > 0.3 to maintain SaO_2_ in the range 88–93%;
- Radiological signs (pulmonary hypoinflation, air bronchogram, hypodiafania of lung fields).

Patients were excluded because of the presence of severe comorbidities, namely: major congenital anomalies, major surgical diseases, genetic-metabolic congenital syndromes.

The following baseline data were collected: antenatal maternal steroids administration, sex, birth weight, gestational age, radiological findings, oxygen saturation (SaO_2_), FiO_2_ requirements, *Silverman score*, resuscitation in delivery room, caffeine prophylaxis and need for surfactant administration.

Newborns were initially stabilized in delivery room with n-CPAP set at 5 cmH_2_O and positive pressure ventilation of 20–25 cmH2O. No sustained ventilation was used, accordingly to 2010 AAP guidelines [16].

When intubation at birth was not needed, neonates were transferred to the Neonatal Intensive Unit on n-CPAP (5 cmH_2_O) during transportation.

At the NICU, all newborns not intubated and ventilated, were started on HFNC as primary respiratory support. HFNC flow rates vary between 4 and 10 L/min titrated according to newborn clinical conditions.

We start at a flow rate of 6 L/min, modulating according to oxygen requirement, CO2 retention or work of breathing, as reported in mechanistic literature [17]. According to our weaning protocol, we reduce flow rates by 0.5 L/min 12 hourly, if required FiO_2_ was lower than 30%. Respiratory rate, work of breathing, desaturations, apneas and bradycardia have been recorded during weaning. The duration of nHFT, i.e. number of days, was also registered.

Chest radiograms were acquired at bedside with a portable device (PHILIPS PRACTIX 33 PLUS MOBILE RADIOGRAPHY®) and classified according to the literature [18].

A prophylactic caffeine citrate therapy (Peyona® CHIESI Farmaceutici, Italy, loading dose of 20 mg/kg and maintenance dose of 5 mg/kg per day) was routinely started early (within 2 h of age) in our cohort of preterm infants < 34 weeks GA and given until 34 to 36 weeks corrected gestational age, if free of any apnea episodes for at least one week [19].

Surfactant (*Curosurf® CHIESI Farmaceutici, Italy*), 200 mg/kg, was administered during nHFT, by INSURE technique, with no pre-medication, if required FiO_2_ was > 35% to achieve SaO_2_ 85–93% [20]. They were immediately extubated thereafter and reassumed to NHFT.

The aim of the study was to determine the effectiveness of nHFT as primary and unique respiratory support, hence neither n-CPAP nor other types of NIV were considered as an alternative support in case of failure.

Therefore, to assess the success rate and the safety of nHFT in our population, the following outcomes were evaluated:

- Percentage of patients that did not need mechanical ventilation within 72 h after the start of nHFT, due to one or more of the following:
  - ▪ arterial or arterialized capillary blood gas analysis showing pH < 7.2 and/or pCO_2_ > 70 mmHg;
  - ▪ FiO_2_ > 40% after surfactant administration to maintain SaO_2_ 85–93%;
  - ▪ more than 4 episodes of apneas with spontaneous recovery within 1 h, or more than 2 episodes requiring IPPV within 1 h.
- Rate of Bronchopulmonary dysplasia (BPD), according to Bancalari et Jobe classification [21]
- Rate of adverse events
  - ○ Air leaks (pneumothorax/pneumomediastinum)
  - ○ Nasal trauma (ulcerations, granulations and vestibular stenosis, and necrosis of the columella)
- Rate of neonates presenting the following diagnoses
  - ○ Late onset sepsis (LOS)
  - ○ Intraventricular hemorrhage (IVH) or periventricular leukomalacia (PVL)
  - ○ Retinopathy of prematurity (ROP)
  - ○ Necrotizing enterocolitis (NEC)
  - ○ Hemodynamically-significant patent ductus arteriosus (PDA) requiring a pharmacologic treatment
- Deaths

Data of total parenteral nutrition duration, time to reach full enteral feeding (defined as a daily intake of ≥140 mL/kg/day), time to full suckling feeds and total length of admissions, were also collected to evaluate indirect advantages.

Data were gathered from medical records of patients admitted to the Neonatal Intensive Care Unit (NICU) of the University of Bari from January 2014 to December 2016.

The database was uploaded as an Ms. Excel spreadsheet and data were analyzed by Stata MP11 software.

Data were presented using standard descriptive statistics: categorical variables were reported as percentages, whereas quantitative variables were described as means ± standard deviations. Chi-squared test was used to perform comparisons between percentages. Test t-student was used to perform comparisons between means in independent variables normally distributed, whereas Wilcoxon rank sum test was used to perform comparisons between means in independent variables non-normally distributed and not normalizable.

For quantitative variables the analysis of normality was carried out; variables non-normally distributed have been normalized using logarithms.

Statistical analysis determined significant factors (maternal and neonatal characteristics) related to outcomes (failure of high-flow nasal cannula therapy, duration and settings of high-flow nasal cannula therapy, hospitalization and time to reach full enteral feeding).

For qualitative variables, in logistic regression we calculated the adjusted Odds Ratio (aOR) value with a 95% confidence interval using the z-score test.

For quantitative variables, in linear regression we calculated the correlation coefficient with a 95% confidence interval using the student’s t-test. In all analyses, a *p*-value of < 0.05 was considered to be statistically significant.

## Results

### Population

**Fig. 1:**
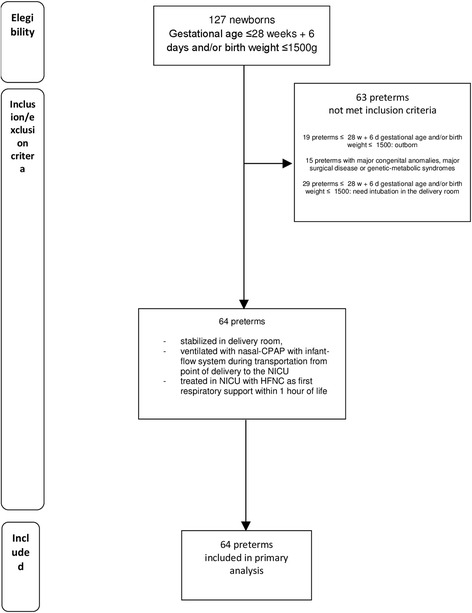
Flow diagram for the patient recruitment in this study

Between January 2014 and December 2016, 838 preterm newborns were admitted to the Neonatal Intensive Care Unit of the University of Bari. Of these, according to inclusion/exclusion criteria, 64 preterms were eventually included (Fig. 1).

Demographics at baseline are described in Table 1.

**Table 1:** Demographic characteristics of newborns enrolled

Demographics characteristics	Results
Mothers	
Age, m (DS)	33.7 (±5,5)
Primigravida, n (%)	36 (56%)
Antenatal glucocorticoids (either complete or incomplete), n (%)^a^	54 (84%)
Cesarean Section, n (%)	56 (87%)
Multiple birth, n (%)	24 (37%)
High risk pregnancy, n (%)^b^	32 (50%)
Newborns	
Gestational age (GA), weeks, m (DS)	29.4 (±2,1)
Distribution per week of gestational age (GA	
26 weeks GA, n (%)	3 (4.5%)
27 weeks GA, n (%)	10 (16%)
28 weeks GA, n (%)	13 (20%)
29 weeks GA, n (%)	7 (11%)
30 weeks GA, n (%)	9 (14%)
31 weeks GA, n (%)	8(12.5%)
32 weeks GA, n (%)	10 (16%)
33 weeks GA, n (%)	3 (4.5%)
34 weeks GA, n (%)	0 (0%)
35 weeks GA, n (%)	1 (1.5%)
Birth weight, g, m (DS)	1166 (±241)
VLBW, n (%)	49 (76%)
ELBW, n (%)	15 (23%)
SGA, n (%)	18 (28%)
Males, n (%)	32 (50%)
Apgar score at 5 min, m (DS)	8.7 (±0,3)
Neonatal resuscitation, n (%)^c^	55 (85%)
Silverman score at enrollment, m (DS)	5.7 (±1,0)
FiO_2_ at enrollment, m (DS)	26.3 (±4,8)
PCO_2_ at enrollment, m (DS)	46.9 (±7,6)
pH at enrollment, m (DS)	7.2 (±0,06)

^a^Antenatal glucocorticoids are medication given to pregnant women expecting a preterm birth that reduce newborn mortality and RDS

^b^High risk pregnancy namely clinically diagnosed chorioamnionitis, prolonged premature rupture of membranes greater than 18 h, preeclampsia, and placental abruption

^c^Oxygen Therapy and Positive pressure ventilation with face mask

Twenty-nine preterm neonates met GA and birth weight inclusion criteria, but were excluded because intubated in the delivery room. Those neonates featured a lower mean GA (26.1 ± 2.5), a lower mean BW (872.2 ± 348.9) and a worse mean Apgar score at 5 min (7.4 ± 1.4) than the enrolled neonates (*p* value < 0.05, data not shown).

Gestational age of neonates started on HFNC ranged between 26^+2^ and 35^+3^ weeks with a median of 29 weeks (DS = ±2.1). Birth weight ranged between 650 and 1495 g with a mean value of 1166 ± 241 g. Males and females were equally represented.

The mean Silverman Anderson Score at the start of nHFT was 5.7 ± 1.0 with a mean FiO_2_ requirement of 26.4 ± 4.8%.

According to our protocol 25 (39.1%) newborns required surfactant administration during treatment. These subgroup median gestational age was 28.5 with a mean birth weight of 1110 ± 232 g.

During NHFT, the mean value of the maximum flow rates reached 7.3 ± 0.9 l/min, whereas the mean value of the maximum FiO_2_ was 28.1 ± 6.5%. Overall, removing patients who needed mechanical ventilation due to NHFT failure, the median duration of treatment was 72.5 h.

### Primary outcome

The overall success rate, i.e. no mechanical ventilation within 72 h after nHFT, was 93.7% (IC 95%: 85% – 100%). nHFT failed in 4 newborns (median GA = 27.5wks, BW = 965 ± 169 g) at a mean postnatal age of 13.5 ± 9.9 h: three of these required surfactant administration during nHFT.

In subgroup analysis, for newborns with GA < 29 weeks, the success rate was 88.5% (*Χ* ^2^ = 2.1; *p* = 0.295; IC 95%: 70% – 98%) and in ELBW newborns, the success rate was 86.7% (*Χ* ^2^ = 1.7; *p* = 0.232; IC 95%: 60% – 98%).

### Secondary outcomes

BPD was diagnosed in 17 newborns (26.6%; IC 95%: 16% – 39%), three of whom (17.6%; IC 95%: 4% – 43%) also needed mechanical ventilation.

In subgroup analysis, BPD was diagnosed in 53.9% (IC 95%: 33% – 73%) of newborns with GA < 29 weeks, in 53.3% (IC 95%: 27% – 79%) of ELBW newborns and in 11.1% (*Χ* ^2^ = 3.1; *p* = 0.117; IC 95%: 0.01–0.36) of SGA. BPD occurred more frequently in newborns with a GA < 29 weeks compared to those of GA ≥ 29 weeks (*Χ* ^2^ = 16.7; *p* = 0.000), and in newborns with a birth weight < 1000 g compared to those weighing ≥ 1000 g (*Χ* ^2^ = 7.2; *p* = 0.007). Neither air leaks nor nasal injury were recorded and no exitus occurred.

Regarding morbidities LOS overall incidence was 16.1%. It occurred respectively in 28% of newborns with GA < 29 weeks (*Χ* ^2^ = 4.4; *p* = 0.07), in 40% of ELBW (*Χ* ^2^ = 8.3; *p* = 0.009) and in 11.1% of SGA (*Χ* ^2^ = 0.5; *p* = 0.709). ROP, hemodynamically-significant PDA and NEC each occurred in 7.8% (IC 95%: 3% – 17%), 9.4% (IC 95%: 4% – 19%), 1.6% (IC 95%: 0% – 8%) of newborns. No cases of IVH or PVL were reported.

Mean duration of total parenteral nutrition was 13.0 ± 9.3 days. Mean time to reach full enteral feeding was 14.6 ± 9.4 days and the mean time to full suckling feeds was 32.1 ± 15.0 days. Duration of hospitalization was 52 ± 20 days. Data on nHFT related outcomes are presented in Table 2.

**Table 2:** Primary and secondary outcomes

Outcomes	Results
Short-term treatment failure (%)	
Total, n (%)	4 (6.3)
< 29 week, n (%)	3 (11.5)
ELBW, n (%)	2 (13.3)
SGA, n (%)	0 (0.0)
Long-term treatment failure (%)	
Total, n (%)	17 (26.6)
• Mild BDP	13 (20%)
• Moderate BDP	3 (4.5%)
• Severe BDP	1 (1.5%)
< 29 week, n (%)	14 (53.9)
ELBW, n (%)	8 (53.3)
SGA, n (%)	2 (11.1)
Adverse event, morbidity, mortality (%)	
Barotrauma and nasal ulceration, n (%)	0 (0.0)
Late onset sepsis, n (%)	10 (16.1)
IVH, n (%)	0 (0.0)
ROP, n (%)	5 (7.8)
NEC, n (%)	1 (1.6)
PDA, n (%)	6 (9.4)
Exitus, n (%)	0 (0.0)
Duration of HFNCT, mean (DS), h	141.1 (±181.1)
Median,	72.5
Min-max	12–720
Maximum flow rates, mean (DS), lpm	7.3 (±0.9)
Median,	7,5
Min-max	4,5–10
Maximum FiO2, mean (DS), %	28.1 (±6.5)
Median,	25,5
Min-max	21–50
Parenteral nutrition duration, mean (DS), days	13.0 (±9.3)
Median,	10
Min-max	2–42
Time to reach full enteral feeding, mean (DS), days	14.6 (±9.4)
Median,	11
Min-max	6–49
Time to bottle feed, mean (DS), days	32.1 (±15.0)
Median,	33
Min-max	7–85
Length of hospitalization, mean (DS), days	52.4 (±20.0)
Median,	49
Min-max	22–94

We also evaluated the relationship between nHFT duration and several maternal and neonatal factors (birth weight, gestational age, weight category for gestational age, delivery mode, gender, maternal age, multiple birth, maternal antenatal steroids, high risk pregnancy, early and late onset sepsis).

In simple linear regression, gestational age (coef. = −0.30; *t* = 4.4; *p* = 0.000; IC 95%: -0.43 – -0.16), birth weight (coef. -0.001; *t* = 2.2; *p* = 0.034; IC 95% -0.0027 – -0.0001), antenatal glucocorticoids (coef. = 0.96; t = 2.2; *p* = 0.029; IC 95%: 0.103–1.820) were positively related to nHFT duration.

In multiple linear regression, only gestational age (coef. = −0.26; *t* = 3.3; *p* = 0.002; IC 95%: -0.42 – -0.10) was positively related to nHFT duration.

Short and long time NHFT failure as well as nHFT duration are not related to FiO_2_ requirements at baseline (*p* > 0.05).

Lower gestational age neonates are likely to have a higher flow rate max (coef. = −1.93; *t* = 2.7; *p* = 0.010; IC 95%: -3.38 – -0.47) and a higher FiO_2_ requirement (coef. = −0.90; *t* = 2.1; *p* = 0.038; IC 95%: -1.75 – -0.05).

Adjusted logistic regression showed that long term failure was positively related to lower gestational age (aOR = 0.43; z = 2.2; *p* = 0.025; IC 95%: 0.20–0.91).

## Discussion

Over the past several years there has been a growing interest in non-invasive ventilation and nHFT as respiratory support in newborns, despite limited data about its safety and effectiveness in the treatment of extremely preterm newborns [22].

Our study on the efficacy and safety of NHFT, as first respiratory support, in preterm newborns with RDS follows other reports [12, 14, 15, 17–19].

Overall, our success and failure rate of nHFT seem comparable with other randomized clinical trials, assessing NHFT as the primary approach to RDS in newborns greater than 29 weeks GA, compared to nCPAP treatment [23, 24].

Furthermore, this is the first study in newborns with GA < 29 weeks and ELBW (< 1000 g), showing a subgroup success rate of 88.5% and 86.7% respectively. In addition, our data showed that lower gestational ages predict NHFT duration and its long-term failure.

Several studies, although conducted for different purposes and enrolling cohorts of either VLBW or very/extremely preterm neonates, reported intubation rates in newborns treated with nCPAP after birth, varying from 12.2% to 52.3% [25–30].

Our intubation rate (6.3%) was much lower so that NHFT seems comparable to nCPAP to avoid the need for mechanical ventilation.

Our mean flow rates (7.3 ± 0.9) was higher than other reports [19, 20], suggesting that NHFT high flow rates could be used even in very preterm newborns without increasing short term failure rate. However, we have had a remarkable rate of late failure (BPD) in newborns with GA < 29 weeks and ELBW compared to other studies using nCPAP [31].

Although the data from our retrospective study seem to support the use of NHFT as primary treatment for preterm newborns with mild respiratory distress, the recent multicentre, randomized HIPSTER trial showed significantly higher rates of treatment failure in newborns primarily treated with NHFT than nCPAP, so that nCPAP should be recommended as first-line treatment, even though intubation rates did not differ between NHFT and nCPAP [32].

In preterm newborns treated with nCPAP, the injuries of nasal mucosa or external nares have been reported [33]. In our cohort, no events of nasal trauma occurred, even in the smallest and extreme preterm newborns.

These data are consistent with those published in a recent meta-analysis, which found a lower incidence of nasal trauma in preterm newborns treated with NHFT compared with other forms of NIV [6].

Furthermore, time to reach both full enteral (14.6 ± 9.4 days) and full suckling feeds (32.1 ± 15.0 days) were lower in our cohort compared to those reported in VLBW newborns less than 30 weeks gestational age [34].

These two outcomes are strictly related to the lower duration of parenteral nutrition (13.0 ± 9.3), the lower rate of late onset sepsis (16.1%), and the shorter hospital stay (52.4 ± 20 days), compared to others [34, 35]. We can speculate that these outcomes are all indirect benefits of NHFT.

We acknowledge several limitations of this study: the retrospective design and the lack of a control group preclude the possibility to make strong assumptions from the presented data on a relatively small sample size.

Our outcome data could be influenced by a large number of SGA newborns (% of the whole sample), but the calculated short term failure rate, with exclusion of SGA newborns, is 8,7% (compared to 6.3%) while the long term failure rate is 32.6%, compared to 26.6% of the entire group (< 29 wks and/or < 1500 g).

## Conclusions

This retrospective study shows that use of High Flow Nasal Cannula Therapy might be feasible and safe as first respiratory support for mild Respiratory Distress Syndrome (RDS) in preterm newborns with a mean gestational age of 29 weeks.

Further well-designed, prospective and randomized trials are needed before to confirm and recommend the use of HFNC in the treatment of respiratory distress of preterm infants.

## Abbreviations

BDP: Bronchopulmonary dysplasia
BW: Birth Weight
ELBW: Extremely low birth weight infants
GA: Gestational age
IVH: Intraventricular hemorrhage
MV: Mechanical ventilation
nCPAP: Nasal continuous positive airway pressure
NEC: Necrotizing enterocolitis
nHFT: Nasal high flow therapy
NICU: Neonatal intensive care units
NIV: Non invasive ventilation
PDA: Patent ductus arteriosus
PVL: Periventricular leukomalacia
RDS: Respiratory distress syndrome
ROP: Retinopathy of prematurity
SGA: Small for gestational age
VLBW: Very low birth weight infants
